# New species of *Homidia* (Collembola, Entomobryidae) from eastern China with description of the first instar larvae

**DOI:** 10.3897/zookeys.152.1455

**Published:** 2011-12-08

**Authors:** Zhi-xiang Pan, Shi-di Shi, Feng Zhang

**Affiliations:** 1 School of Life Sciences, Taizhou University, Linhai, Zhejiang 317000, China; 2 School of Life Sciences, Nanjing University, Nanjing, Jiangsu 210093, China

**Keywords:** new species, first instar, scanning electron microscope, chaetotaxy, China

## Abstract

Morphology of the first instar larvae of Collembola has considerably taxonomical and phylogenetic significance. We describe the first instar larvae for the first time in *Homidia*. External morphology of first instar larvae and adults of *Homidia jordanai* sp. n. is described based on observations under light and scanning electron microscopes. Most organs of adults bear considerably more setae than the first instar larvae; in addition, first instar larval *Homidia* lack labial seta R, seta on tenaculum, mucronal spine, and dental spines. The new species is characterized by weakly pigmented body, long antennae subequal to body in length, 1+1 inner macrochaetae on Abd. III, few inner macrochaetae on posterior Abd. IV, and spiny and short seta pi on dental base. Differences between new species and other two similar ones, taxonomical significance of the first instar larvae and the position of *Homidia* are also discussed.

## Introduction

In epimetabolic Collembola, the number of setae and pigmentation often change after moult (Christiansen 1992). Morphology of the first instar larvae has considerably taxonomical and phylogenetic significance (Szeptycki 1979), but it has been rarely studied. *Homidia* is the most widespread entomobryid genus in East Asia, particularly Japan, Korea and China. The genus is characterized by presence of spines on inner edge of dens, “eyebrow” macrochaetae on anterior part of Abd. IV of adults, ommatidia 8+8, mucro bidentate with subapical tooth much larger than the apical one, and absence of body scales. The chaetotaxy of first instar larvae (primary chaetotaxy) in *Homidia* has never been described.

In the present paper, the primary chaetotaxy of *Homidia* was studied for the first time based on *Homidia jordanai* sp. n., from East China. Many other morphological details are showed under scanning electron microscope.

## Material and methods

Alcohol preserved young and adult specimens, were cleared in lactic acid, mounted on microscope slides in Marc André II solution, and studied using Leica DM2500 and Nikon 80i microscopes. Few specimens, coated with platimum under vacuum conditions, were observed under scanning electron microscope. Photographs were taken with Leica AL2 and Nikon SMZ1000 microscopes using a mounted Nikon DS-Fi1 camera and Hitachi S4800 scanning electron microscope, numbers and letters added with photoshop CS2 (Adobe Inc.), all length data measured with Nikon NIS-Elements Documentation 3.1. First instar larvae were determined by the stability of primary chaetotaxy (e.g. 19/18 common setae on meso-/metathorax, Szeptycki 1979). Cephalic dorsal chaetotaxy is designated after Jordana and Baquero (2005) and Soto-Adames (2008), interocular setae after (Mari-Mutt (1979, 1986), labial palp setae after Fjellberg (1998), labial setae after Gisin (1964), dorsal chaetotaxy after Szeptycki (1979).

Abbreviations. Th. –thoracic segment, Abd. –abdominal segment, Ant. –antennal segment, ms –microsensillum/a, s –common sensillum/a, mac –macrochaeta(e), mic –microchaeta(e), SEM –scanning electron microscope, LM –light microscope, Tita I–III –tibiotarsus of fore, mid, and hind legs.

## Results

### Homidia jordanai sp. n.

urn:lsid:zoobank.org:act:7A40BC6A-99BB-4A02-AE92-0899F6556BD8

http://species-id.net/wiki/Homidia_jordanai

Figs 1 [Fig F2] [Fig F3] [Fig F4] [Fig F5] [Fig F6] [Fig F7] [Fig F8] [Fig F9] [Fig F10] [Fig F11] [Fig F12] [Fig F13] –80 Tables 1 [Table T2] –3

#### Holotype.

♂ on slide, Shaoxin City, Zhuji Country, Dongbaihu, Zhejiang Province, CHINA, 29°34.48'N, 120°24.32'E, 3.X.2009, collection number S4014, collected by Zhi-Xiang Pan and Chen-Chong Si, deposited in Taizhou University.

#### Paratypes.

2 ♂, 11 ♀ and 5 larvae on slide, numerous in alcohol, same data as holotype. 5 paratypes (1 ♂, 1 ♀ on slide, 1 larva and 2 adults in alcohol) deposited in School of Life Sciences, Nanjing University and others in Taizhou University, China.

#### Etymology.

Named after the famous Spanish entomologist Jordana Rafael (University of Navarra).

#### Description.

**Adult.** Size. Maximum body length up to 2.3 mm.

**Figures 1–4.**
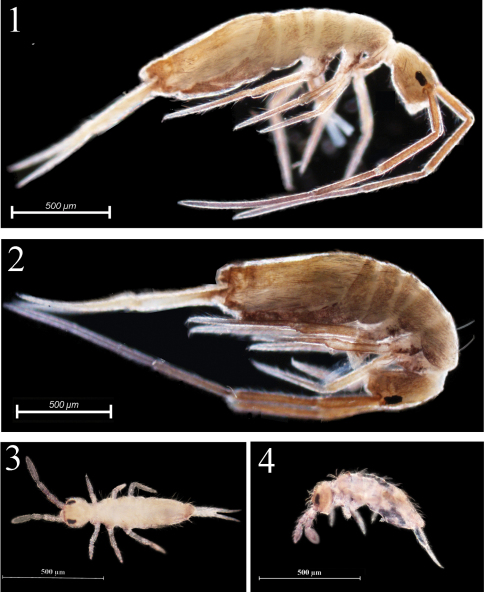
Habitus of *Homidia jordanai* sp. n. **1–2** adults, lateral view **3–4** the first instar larvae **3** dorsal view **4** lateral view.

Habitus. Ground colour pale yellow in alcohol. Body dorsally without pigment. Coxa and trochanter of all legs with weak blue pigment. Eye patches dark. Antennae gradually darker from Ant. III to Ant. IV (Figs 1, 2).

**Figures 5–9. F2:**
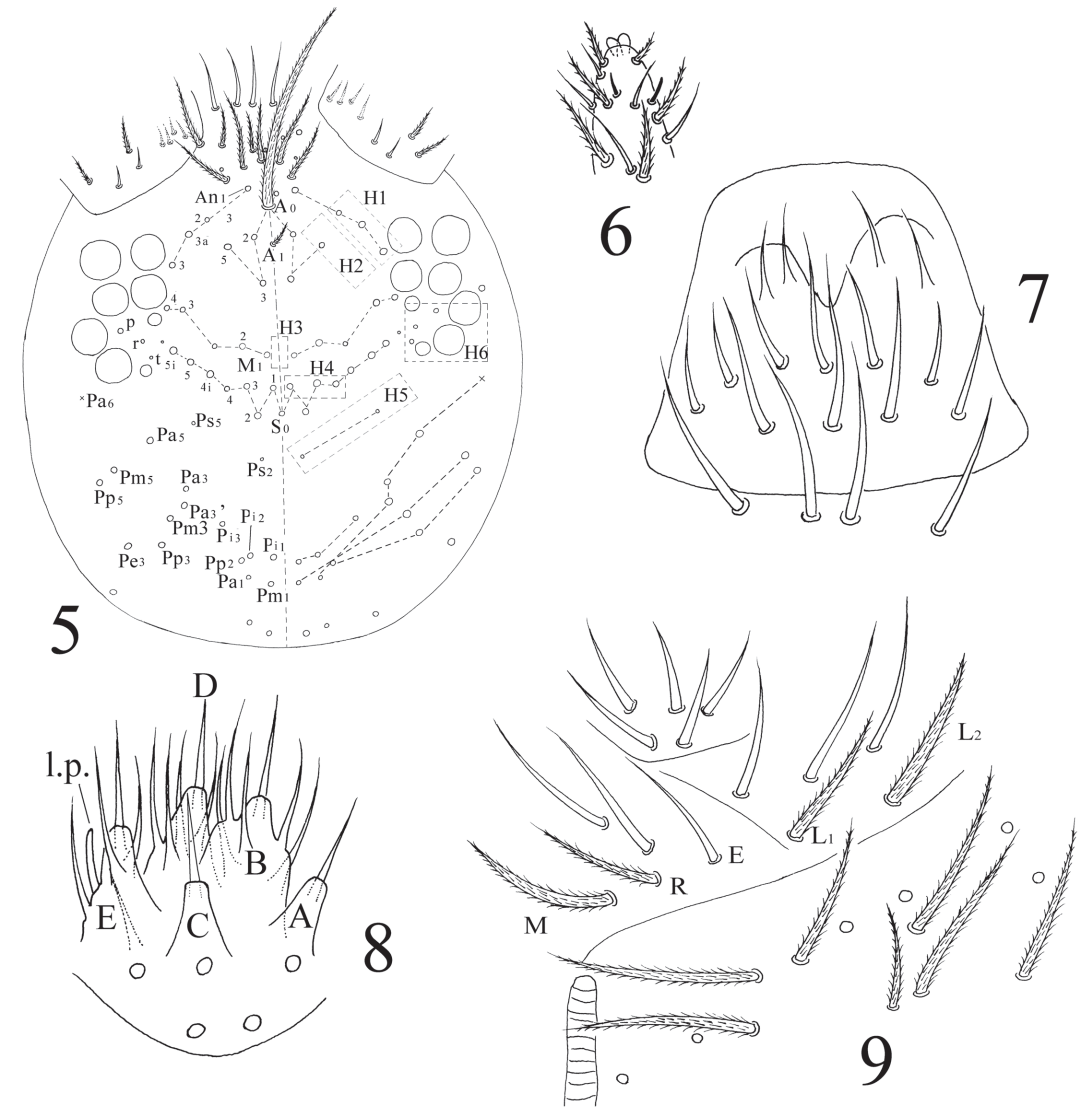
*Homidia jordanai* sp. n. **5** dorsal cephalic chaetotaxy **6** apical bulb of Ant. IV **7** labrum **8** labial palp **9** labial base.

Head. Ommatidia 8+8, G and H smaller than others, and sometimes invisible under LM, interocular setae as p, r, t (Figs 5, 44). Antenna 3.9–4.6 times as long as cephalic diagonal, subequal to body in length, antennal segment ratio as I : II : III : IV = 1 : 1.3–1.5 : 1.3–1.4 : 2.4–3.1. Basal Ant. I with 2 dorsal and 4 ventral spiny setae (Figs 5, 45, 46). Basal Ant. II with 5 smooth setae (Figs 47, 48); distal Ant. II with 4 s (2–3 longer, 1–2 shorter) (Figs 52, 53). Ant. III organ with 2 rod-like s and 3 small s (Figs 54–56); those s also with obvious ridges on surface under SEM. Distal Ant. IV with several types of s (Figs 57–62); apical bulb bilobed (Fig. 6). Dorsal cephalic chaetotaxy with 4An and 7S mac. Clypeus with many ciliate setae (Fig. 5). Labral papillae absent. Prelabral and labral setae as 4/5, 5, 4, all smooth, labium intrusion U-shaped (Fig. 7). Maxillary outer lobe with 1 apical seta, 1 subapical seta and 3 sublobal hairs on sublobal plate; subapical seta slightly longer than apical one (Fig. 49). Labial palp with five papillae A–E, with 0, 5, 0, 4, 4 guard setae, respectively, and 5 smooth proximal smooth setae; lateral process differentiated with blunt tip reaching apex of papilla E (Figs 8, 50, 51). Hyaline plate with 1 main (H) and 2 accessorial (h_1_, h_2_) setae. Setal formula of labial base as MREL_1_L_2_, seta E smooth, others ciliate (Fig. 9).

**Figures 10–14 F3:**
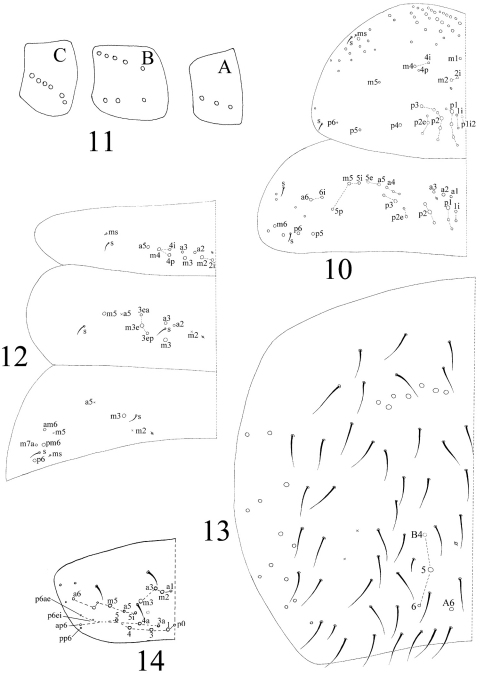
**.** *Homidia jordanai* sp. n. **10** dorsal chaetotaxy of Th. II–III **11** coxal chaetotaxy formula (**A** fore leg **B** mid leg **C** hind leg) **12–14** dorsalchaetotax **12** Abd. I**–**III **13** Abd. IV **14** Abd. V.

**Figures 15–19. F4:**
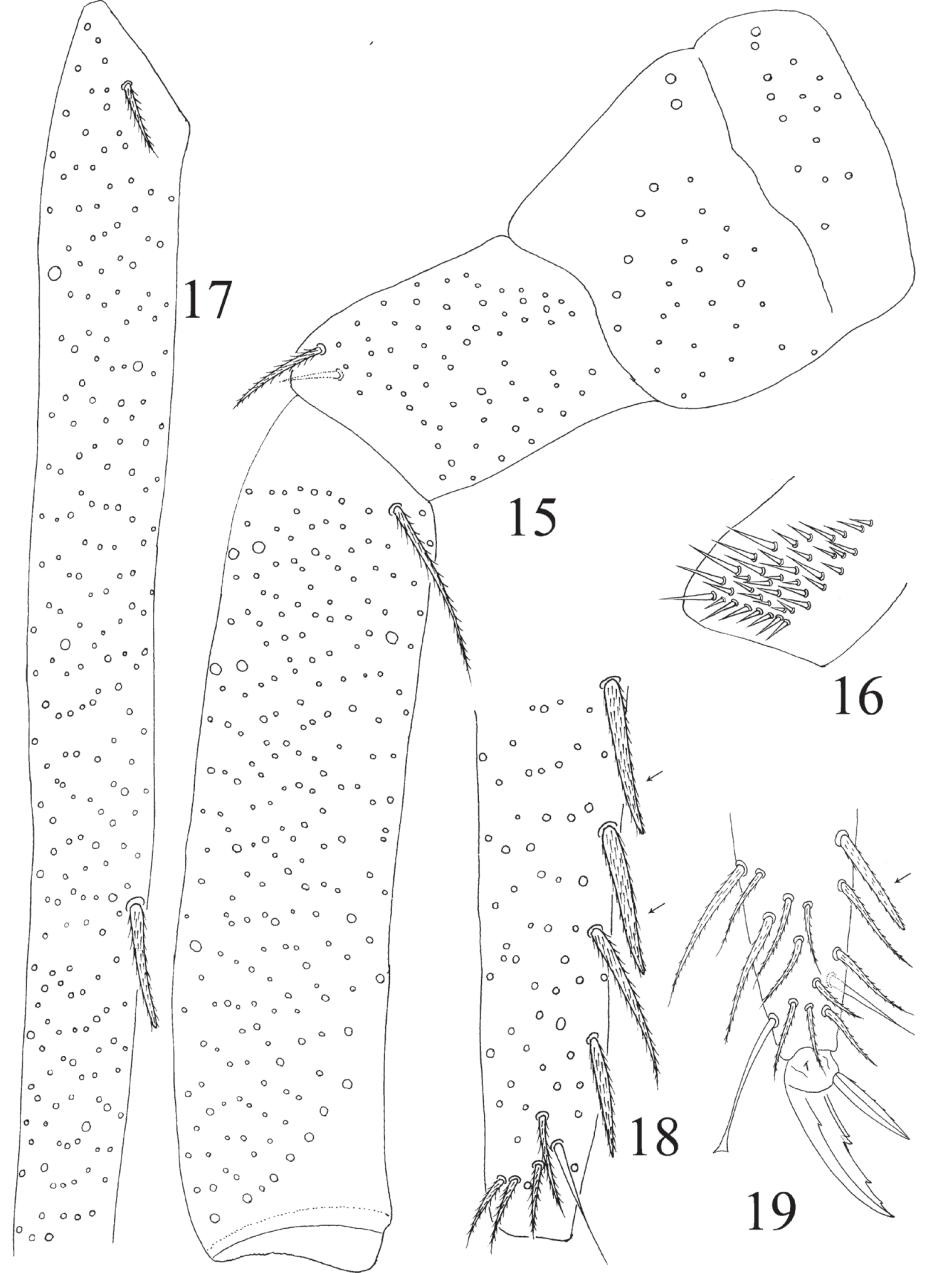
Hind leg of *Homidia jordanai* sp. n. **15** coxa, trochanter and femur **16** trochanteral organ **17** basal Tita **18** apical Tita **19** apical Tita and claw.

Thorax. Complete s-chaetae of dorsal body as 32/223(>47)3 (examined specimens mostly with 47 s-chaetae on Abd. IV, but some lost during preparation), ms as 10/10100. Th. II with 3 medio-medial (m1, m2, m2i), 3 medio-sublateral (m4, m4i, m4p), 17–18 posterior mac and 3 s-chaetae (ms antero-internal to s); seta p1i2 rarely present, seta p6 as mic. Th. III with 29–32 mac and 2 s-chaetae; setae p1i2 and p4 absent (Fig. 10). Numerous setae on hind leg (Figs 15–19); pseudopores on coxa shown in Fig. 63, but their number unclearly seen. Coxal macrochaetal formula as 3/4+1, 2+1/4+2 (Fig. 11). Trochanteral organ with 36–64 smooth spiny setae (Fig. 16). Inner differentiated tibiotarsal setae slightly ciliate, most distal smooth seta present on hind leg (Figs 18, 19, 65). Tenent hairclavate and subequal to inner edge of unguis in length (Figs 19, 66). Unguis with 4 inner, 2lateral and 1 outer teeth, all tiny. Unguiculus lanceolate with outer edge slightly serrate (Fig. 65). Pretarsus with 1 pair of small spines (Figs 19, 67).

**Figures 20–24. F5:**
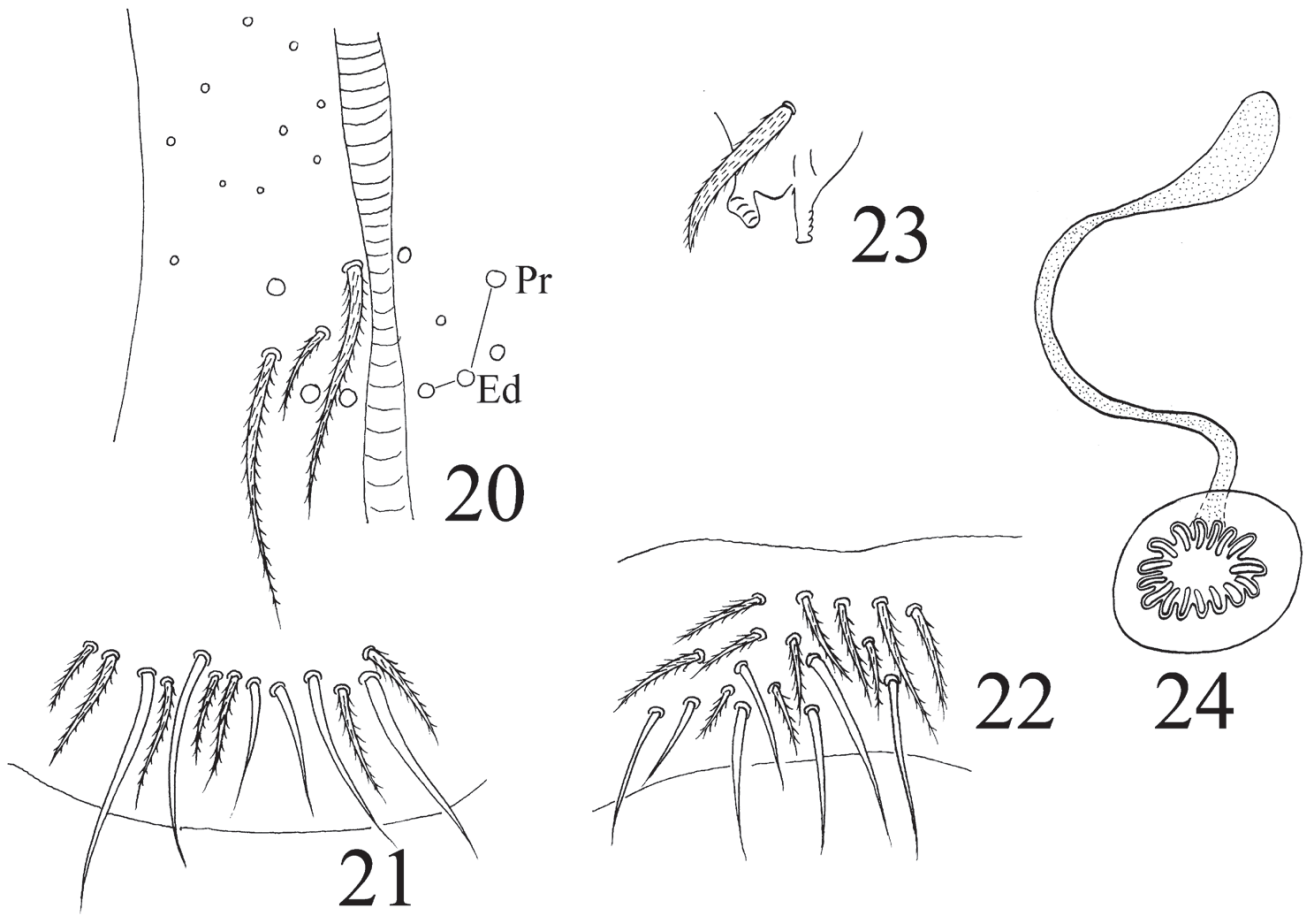
*Homidia jordanai* sp. n. **20–22** ventral tube **20** anterior face **21** posterior face **22** lateral flap **23** tenaculum **24** genital plate and spermary.

**Figures 25–28. F6:**
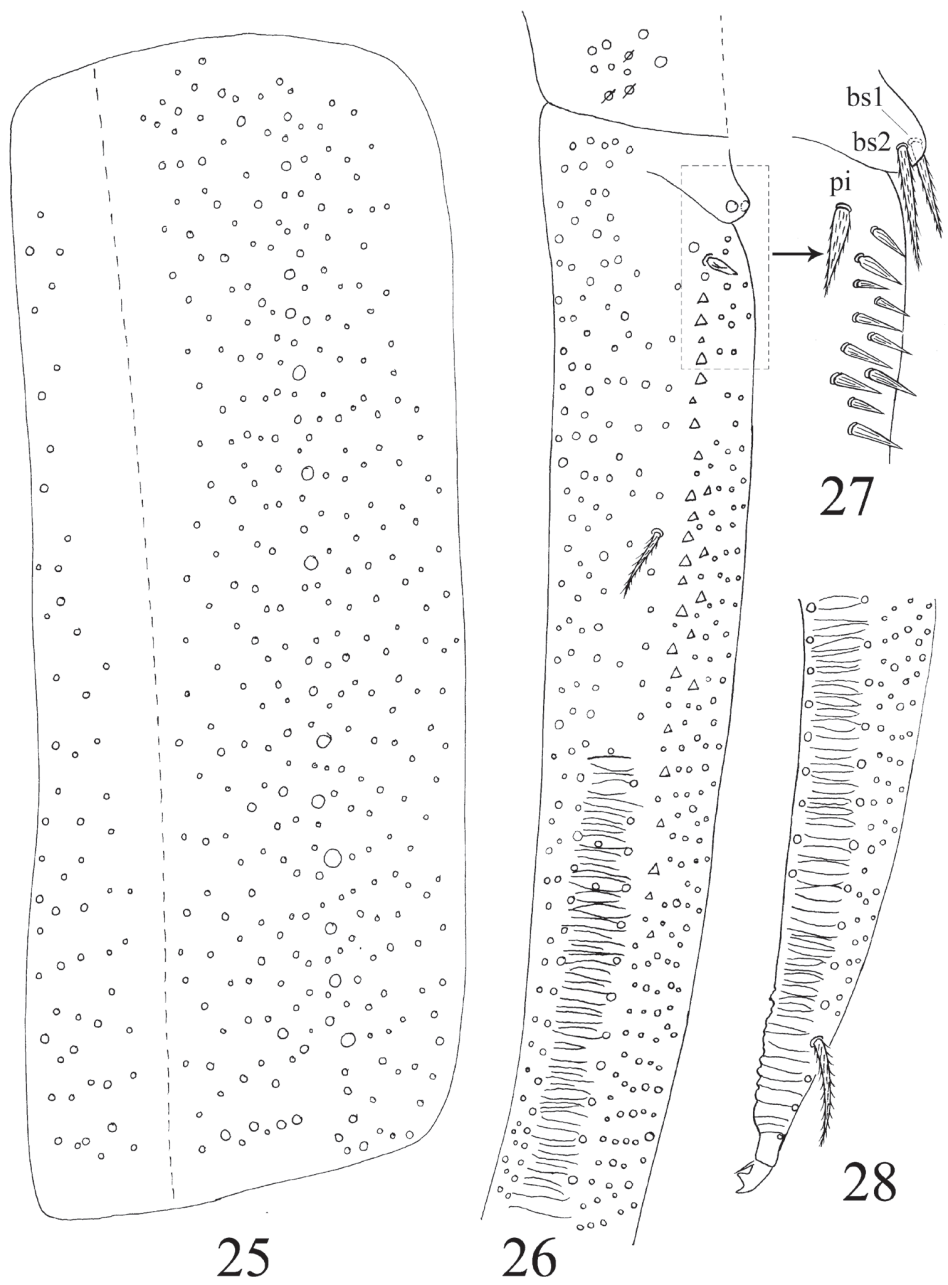
Furcula of *Homidia jordanai* sp. n. **25** manubrium **26** manubrial plaque and base of dens **27** inner side of dens (triangles representing spines) **28** apical dens and mucro.

Abdomen. Abd. I with 9 (a2, a3, m2–4, m2i, m4i, m4p, a5) mac and 2 s-chaetae (ms anterio-external to s) (Figs 68, 69). Abd. II with 6 (a2, a3, m3, m3e, m3ea, m3ep) inner, 1 (m5) lateral mac, and 2 s-chaetae. Abd. III with 1 (m3) inner and 4 (am6, pm6, m7a, p6) lateral mac, and 3 s-chaetae (Fig. 12). Abd. IV with more than 47 s-chaetae (2 of normal length and others elongated), 6–9 mac on anterior part and irregularly arranged in a transverse row; posterior part with 2 (3) (B5 and A6, A6 rarely as mic, B4 sometimes present) mac and 1 (B6) mic (Fig. 13). Abd. V with 3 s-chaetae; m3a absent, a5i sometimes absent (Fig. 14). Anterior face of ventral tube with many ciliate setae, including 4+4 mac, line connecting proximal (Pr) and external-distal (Ed) (Chnd and Li 1997) mac oblique to median furrow(Fig. 20); posterior face with 5 or 6 (median with 1 or 2 small) smooth and numerous ciliate setae (Fig. 21); lateral flap with 6–7 smooth and 10–22 ciliate setae (Figs 22, 74, 75). Furcula shown in Figs 25–28. Manubrial plaque with 3 pseudopores, 2 inner and 5–6 outer ciliate setae (Fig. 26). Dens with 20–40 spines (Figs 26, 27, 77); basal sete (Szeptycki 1973) bs_1_ and bs_2_ spiny and multilaterally ciliate, bs_1_ shorter than bs_2_; proximal-inner seta (pi) spiny, shorter and thicker than bs_1_ and bs_2_ (Figs 26, 27). Mucro bidentate with subapical tooth obviously larger than apical one; basal spine short, with tip only reaching apex of subapical tooth (Figs 28, 76). Tenaculum with 4+4 teeth and 1 large, multilaterally ciliate basal seta (Fig. 23). Genital plate papillate (Fig. 24).

#### The first instar larva.

Size. Body length up to 0.7 mm.

Habitus. Ground colour pale white in alcohol. Eye patches dark. Distal antennae slightly pigmented (Figs 3, 4).

**Figures 29–32. F7:**
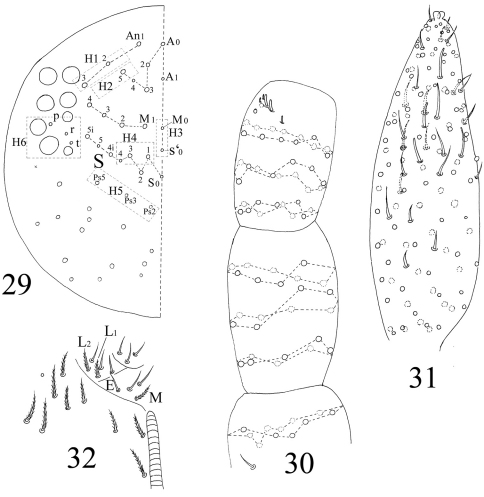
The first instar larval *Homidia jordanai* sp. n. **29** dorsal cephalic chaetotaxy **30** Ant. I–III **31** Ant. IV **32** labal base

Head. Antenna 1.3–1.8 times as long as cephalic diagonal, antennal segment ratio as I : II : III : IV = 1 : 1.4–2.2 : 1.5–2.5 : 3.3–4.3. Ant. I with 11 ciliate and 1 basal spiny setae. Ant. II with 25 ciliate setae. Ant. III with 38 ciliate setae and 5 s-chaetae of Ant. III organ (Figs 30, 78). Ant. IV with numerous ciliate setae and some s-chaetae (Figs 31, 79). Dorsal cephalic chaetotaxy as 3An and 5S mac (Fig. 29). Labium with 3 smooth proximal setae. Setal formula of labial base as MEL_1_L_2_, seta E smooth, all others ciliate (Fig. 32). Ommatidia, Ant. IV apical bulb, interocular setae, labral papillae, labrum, maxillary outer lobe, labial palp, hyaline plate same as adults.

**Figures 33–37. F8:**
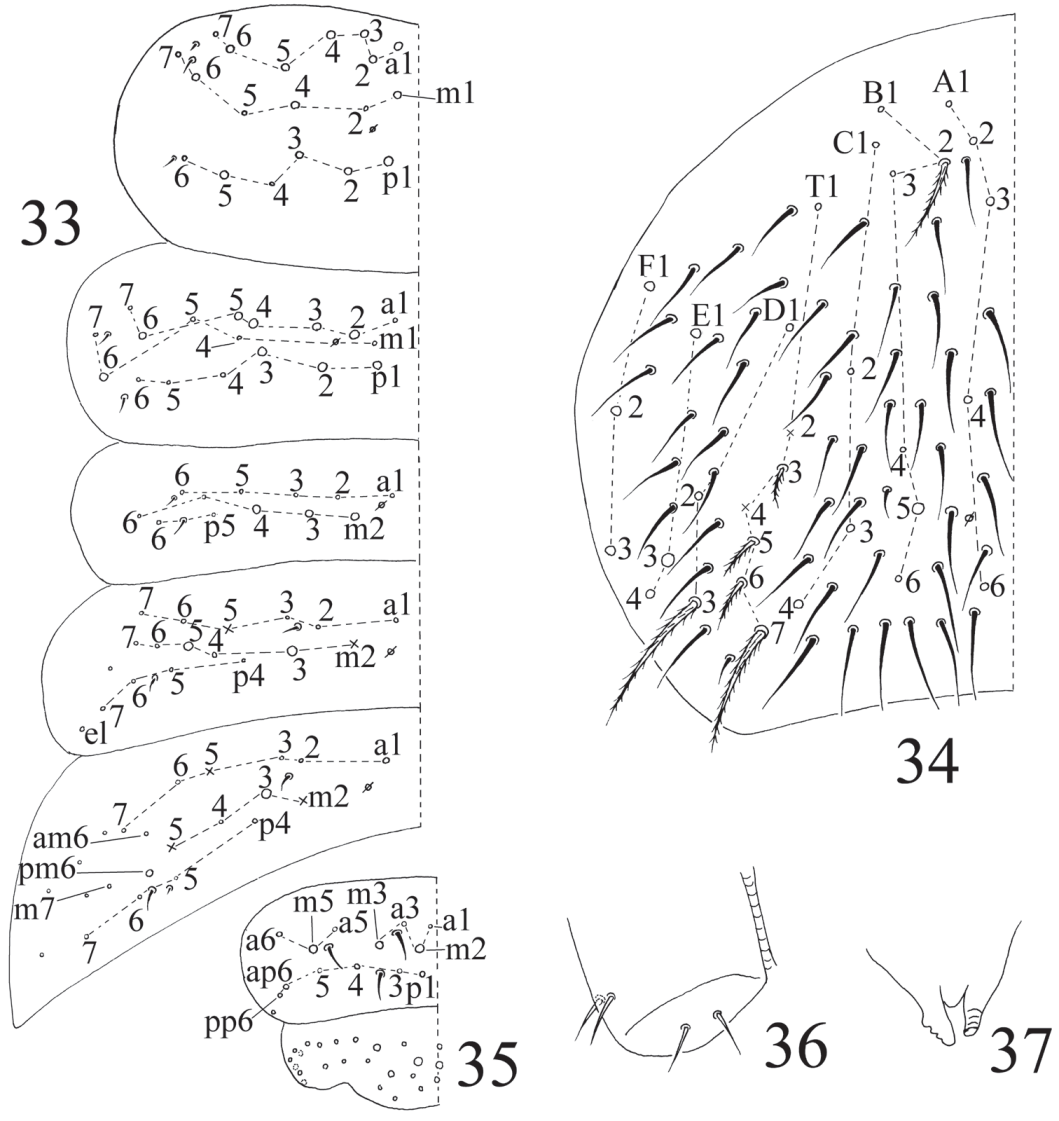
The first instar larval *Homidia jordanai* sp. n. **33–35** dorsal chaetotaxy **33** Th. II–Abd. III **34** Abd. IV **35** Abd. V–VI **36** ventral tube **37** tenaculum.

Chaetotaxy. Complete s-chaetae of body as 32/223(50–53)3, ms as 10/10100. Th. II with 13 (a1–6, m1, m4, m6, p1–3, p5) mac, 6 (a7, m2, m5, m7, p4, p6) mic and 3 s-chaetae (ms anterior to s), m1 rarely as mic. Th. III with 9 (a2–6, m6, p1–3) mac, 9 (a1, a7, m1, m4, m5, m7, p4, p6) mic and 2 s-chaetae. Abd. I with 3 (m2–4) mac, 9 (a1–3, a5, a6, m5, m6, p5, p6) mic and 2 s-chaetae (ms antero-external to s). Abd. II with 2 (m3, m5) mac, 13 (a1–3, a6, a7, m4, m6, m7, p4, p7, el) mic, 1 additional mic on lateral and 2 s-chaetae. Abd. III with 1 (m3) mac central, 13 (a1–3, a6, a7, m4, am6, pm6, m7, p4) mic, 3 s-chaetae and 5 lateral additional mic (Fig. 33). Abd. IV with 2 (B5, E3) mac, 27 (A1–4, A6, B1–4, B6, C1–4, T1, T3, T5, D1–3, E1, E4, F1–3) mic and 50–53 s-chaetae (48–51 elongated and 2 of normal length); setae A5 and E2 absent (Fig. 34). Abd. V with total 14 setae and 3 s-chaetae. Abd. VI with 21+21 setae; 3 on middle line (Fig. 35).

**Figures 38–43. F9:**
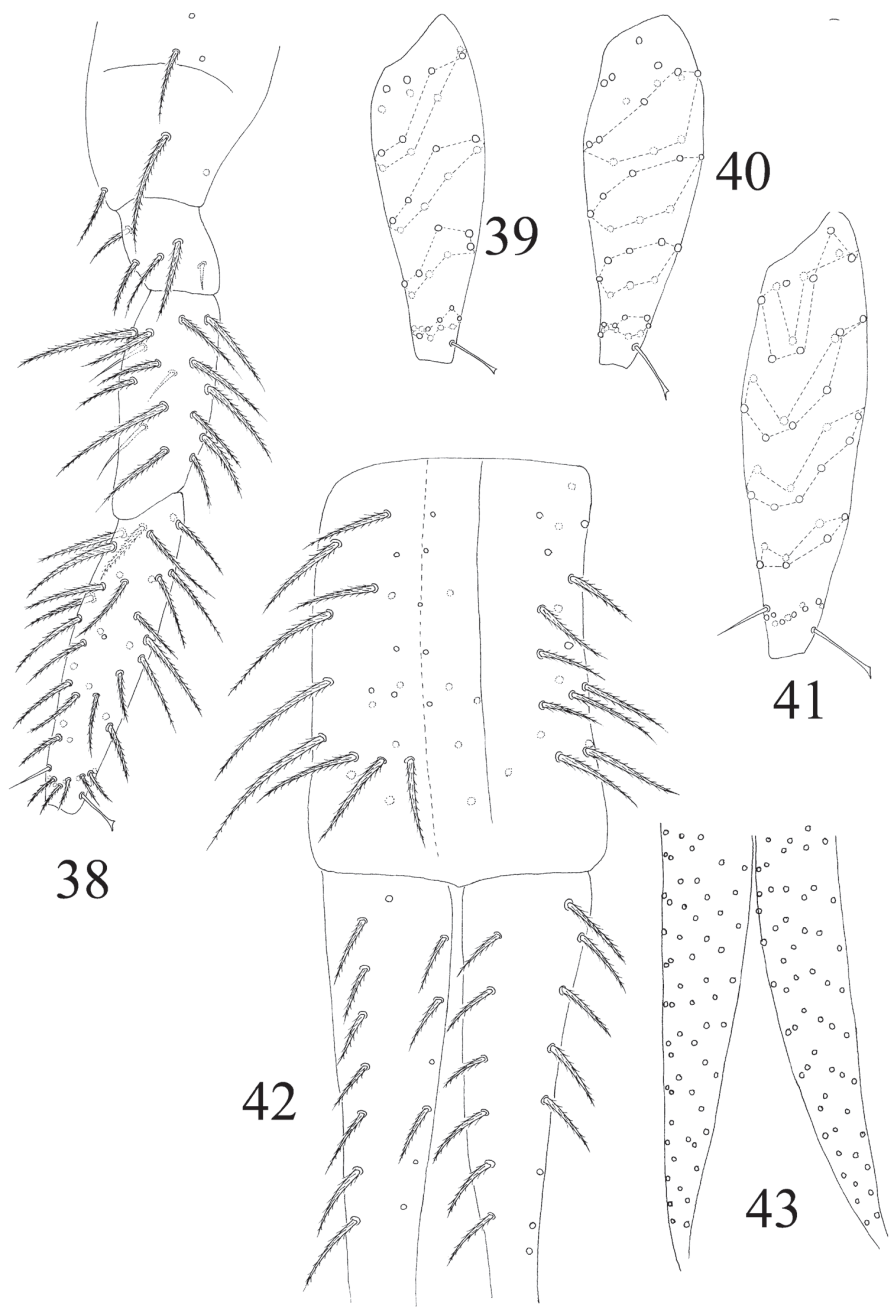
The first instar larval *Homidia jordanai* sp. n. **38** hind leg **39** Tita I **40** Tita II **41** Tita III **42** manubrium and dorsal dens **43** ventral dens.

Leg. Coxa I–III with 2, 3, 5 ciliate setae. Trochanter I–III with 4, 5, 4 ciliate and 2, 1, 0 smooth setae, 1 spine on trochanter III. Femur I–III with 10, 16, 14 ciliate and 3, 1, 3 smooth setae. Tita I–III with 38, 41, 46 ciliate setae and 1 tenent hair respectively, 1 supraempodial seta on Tita III (Figs 38–41). Unguis with 4 minute inner and 2lateral teeth. Unguiculus lanceolate with outer edge slightly serrate (Fig. 80).

Ventral tube. Anterior face without seta; posterior face with 2 apical smooth setae; lateral flap with 2 smooth setae (Fig. 36).

Furcula. Manubrum with 24+24 ciliate setae. Manubrial plaque not seen. Dorsal dens with 14–15 (8 in outer 6–7 in inner row) setae, ventral side with about 55 ciliate setae, inner of dens without dental spine, basal setae (bs_1_ and bs_2_) absent (Figs 42, 43). Mucro bidentate with subapical tooth obviously larger than apical one; basal spine absent. Tenaculum with 4+4 teeth but without setae on corpus (Fig. 37).

#### Ecology.

In the leaf litter of *Cunninghamia lanceolata* (Lambert) and *Dicranopteris dichotoma* (Thunberg).

#### Remarks.

The new species is characterized by weak pigment on dorsal body, long antennae subequal to body in length, labial basal seta L_1_ ciliate, absence of setae m2i2 on Th. II, a1 on Abd. I, a2 on Abd. III, A4–5 on Abd. IV, and dental basal seta pi spiny and shorter than bs. It is similar to *Homidia unichaeta* Pan, Shi & Zhang, 2010 and *Homidia tibetensis* Chen & Zhong, 1998 in colour pattern, cephalic chaetotaxy, labrum, coxal formula, chaetotaxy of Abd. II and claw. However, it can be distinguished from them by the length of antennae, labial setal formula, chaetotaxy on Th. II, Abd. I, Abd. III–IV and seta pi on basal dens. Additional differences are listed in Table 1.

#### Differences between the first instar larvae and adults.

Some characters are principally same in the first instar larvae and adults: ommatidia, interocular setae, Ant. III organ, apical bulb on Ant. IV, labrum and labral papillae, labial palp, maxillary outer lobe, claw, bothriotricha and s-chaetoxic pattern on terga.

Characters that develop after the first instar: s on distal part of Ant. II, smooth setae on base of Ant. II, labial seta R, smooth spiny setae on trochanteral organ, mac on anterior face of ventral tube, seta on corpus of tenaculum, pseudopores on manubral plaque, basal spine on mucro and genital plate.

Chaetotaxy become more complicated during postembryonal development, detailed differences between the first instar larvae and adults (apart from chaetotaxy of body tergites) are listed in Table 2. Tergal chaetotaxy of adults becomes much more complicated than that of primary chaetotaxy. In addition to numerous secondary common mic and mac on terga, some primary mic are transformed into mac: m2, m5 and p4 on Th. II, a1, m5, p5 and p6 on Th. III, a2, a3 and a5 on Abd. I, a2 and a3 on Abd. II, am6 and p6 on Abd. III, A6 and B4 on Abd. IV (homology of lateral setae difficult to determine).

**Figures 44–51. F10:**
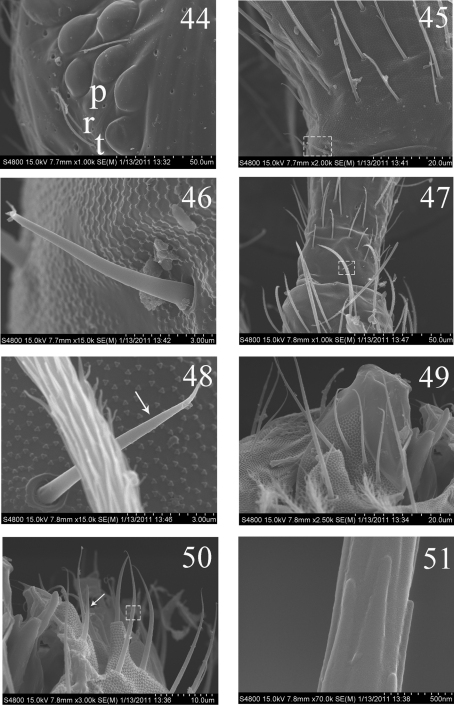
SEM photos of adult *Homidia jordanai* sp. n. **44** eye patches **45** base of Ant. I, dorsal side **46** spiny setae on base of Ant. I, dorsal side **47** joint of Ant. I and Ant. II **48** spiny seta on base of Ant. II **49** maxillary outer lobe **50** labial palp **51** micro-architecture of proximal setae.

**Figures 52–59. F11:**
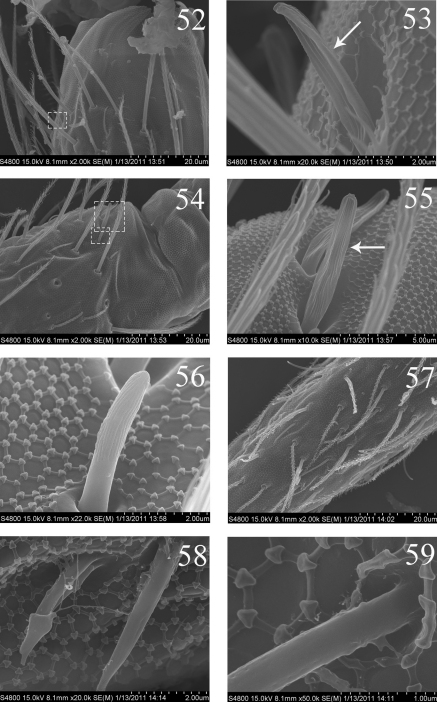
SEM photos of adult *Homidia jordanai* sp. n. **52** distal part of Ant. II **53** s on distal part of Ant. II **54** Ant. III organ **55** internal two s of Ant. III organ **56** external s of Ant. III organ **57** Ant. IV **58–59** s on Ant. IV.

**Figures 60–67. F12:**
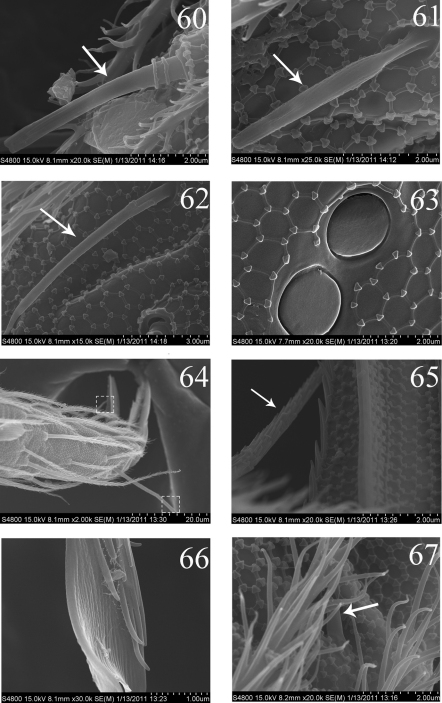
SEM photos of adult *Homidia jordanai* sp. n. **60–62** s of Ant. IV **63** pseudopores of coxa **64** Tita and claw of hind leg **65** supraempodial seta and outer edge of unguiculus **66** distal part of tenent hair **67** spiny seta on pretarsus.

**Figures 68–75. F13:**
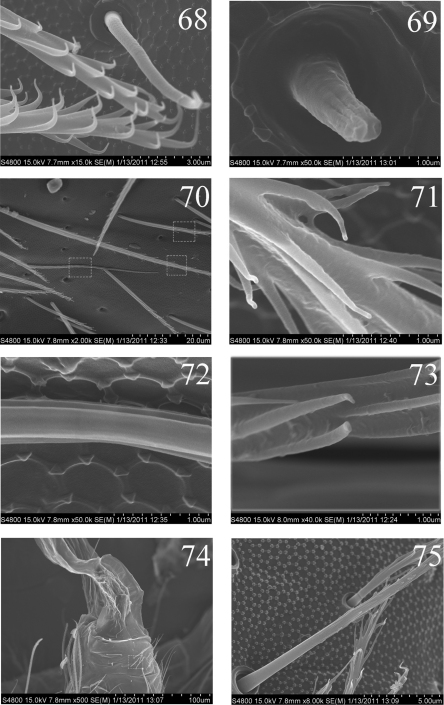
SEM photos of adult *Homidia jordanai* sp. n. **68** lateral s-chaetae on Abd. I **69** lateral ms on Abd. I **70** three types of setae on Abd. IV **71** bothriotrichum on Abd. IV **72** elongate s-chaetae on Abd. IV **73** ciliate seta on Abd. IV **74** distal part of ventral tube **75** two types of setae on lateral flap of ventral tube.

**Figures 76–80.**
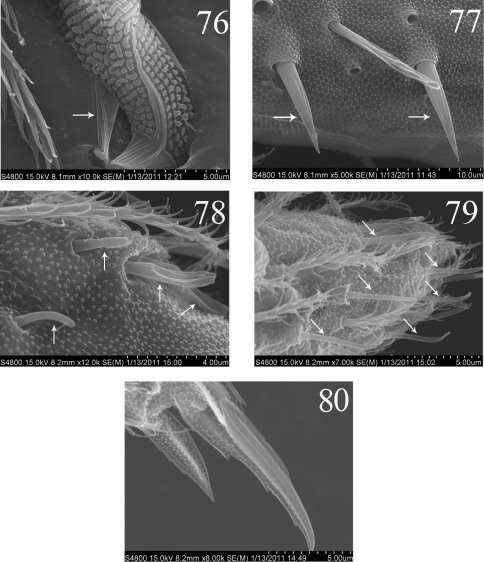
SEM photos of *Homidia jordanai* sp. n. **76–77** adult **76** partial mucro **77** dental spines **78–80** the first instar larva **78** Ant. III organ **79** distal part of Ant. IV **80** distal part of hind leg.

**Table 1.** Main differences between *Homidia jordanai* sp. n. and two similar species.

Characters	*H. j*	*H. u*	*H. t*
Pigment on dorsal tergite	-	-	slight
Antenna length as long as cephalic diagonal	3.9–4.6	1.5–2.5	about 3.5 times
Seta L_1_ on labial base	ciliate	smooth	smooth
Lateral prosess of labial papilla E	reach apex	not reach	?
Seta m2i2 on Th. II	-	+	-
Seta a1 on Abd. I	-	+	+
Seta a2 on Abd. III	-	+	+
Mac on posterior Abd. IV	2 (rarely 3)	1	2
“Eyebrow” setae on anterior Abd. IV	6–9	3–8 (usually 5–7)	10–12
Dental spines	20–40	19–23	39–54
Comparison of dental basal seta in length	bs > pi	pi > bs	bs > pi
Type locality (China)	Zhejiang	Zhejiang	Tibet

*H. j*: *Homidia jordanai* sp. n.; *H. u*: *Homidia unichaeta*; *H. t*: *Homidia tibetensis*; -: absent; +: present;?: the character unclear.

**Table 2. T2:** Differences between the first instar larvae and adults (apart from chaetotaxy of body tergites).

Characters	First instar larvae	Adults
Ground colour	pale white	pale yellow
Cephalic chaetotaxy	3An, 5S	4An, 7S
Spiny setae on basal Ant. I	1	2 dorsal, 4 ventral
S on distal Ant. II	-	4
Proximal setae of labium	3	5
Seta R on labial base	-	+
Coxal macrochaetal formula	2/3/5	3/4+1, 3/4+2
Setae on trochanteral organ of hind leg	1	36–64
Inner tibiotarsal setae	strongly ciliate	thicker and slightly serrate
Setae on anterior face of ventral tube	-	4+4 mac and numerous mic
Setae on lateral flap of ventral tube	2 smooth	6–7 smooth and 10–24 ciliate
Setae on posterior face of ventral tube	2 smooth	5 or 6 smooth and numerous ciliate
Seta on tenaculum	-	1
“Eyebrow” setae on Abd. IV	-	6–9
Setae on manubrium	24+24	more than 24+24
Pseudopores on manubrial plaque	-	3
Ciliate setae on manubrial plaque	-	7–8
Basal setae (bs_1 _and bs_2_) on dens	-	+
The shape of proximal-inner seta (pi)	normal	spiny
Dental spines	-	20–40
Basal spine of mucro	-	+
Genital plate	-	papillate

-: absent; +: present.

**Table 3.** Morphological differences of the first instar larvae among six Entomobyridae species.

tergite	seta	*H. j*	*O. f*	*H. n*	*E. m*	*P. a*	*S. d*
Th. II	m1	mac	mac	mic	mac	mac	mac
	m2	mic	mac	mic	mic	scale	mac
	p5	mac	mac	mic	mic	mac	mac
Th. III	a2	mac	mic	mic	mic	mic	mac
	a3	mac	mic	mic	mic	mic	mic
	a4	mac	mac	mic	mic	mac	mac
	m1	mic	mic	mic	mic	-	mic
	m2	-	-	-	-	mac	-
Abd. I	m2	mac	mac	mac	mic	mac	mac
	m4	mac	mac	mic	mac	mac	mac
	m6	mic	mic	mic	mic	mic	mic
Abd. II	a2	mic	mac	mic	mic	mac	mac
	m5	mac	mac	mic	mac	mac	mac
Abd. III	pm6	mac	mac	mac	mac	mac	mac
	p6	mic	mic	mic	mac	mic	mac
Abd. IV	A4	mic	-	-	mic	-	-
	A5	-	-	-	-	mic	mic
	B4	mic	-	-	mic	mic	mac
	B5	mac	mic	mic	mac	mac	mac
	B6	mic	mic	-	mic	mic	mic
	E2	-	mac	mic	-	mic	mic
	E3	mac	-	-	mac	mic	mac

*H. j*: *Homidia jordanai* sp. n.; *O. f*: *Orchesella flavescens* (Bourlet, 1839); *H. n*: *Heteromurus nitidus* (Templeton, R in Templeton, R & Westwood, J. O, 1836); *E. m*: *Entomobryoides myrmecophila* (Reuter, 1884); *P. a*: *Pseudosinella alba* (Packard, 1873); *S. d*: *Seira dowlingi* (Wray, 1953); -: absent.

## Discussion

### Taxonomical significance of the first instar larvae and the position of the genus *Homidia*

The adult chaetotaxy of Entomobryidae exhibits marked differences among genera or species. Szeptycki (1972) found that the primary chaetotaxy of Entomobryomorpha was almost identical in number and position. Later, he (1979) studied the ontogeny of tergal chaetotaxy of the representative Entomobryidae genera and its preliminary phylogenetic significance at higher level of hierarchy with four subfamilies included in Entomobryidae. Subsequent authors (Rusek 2002; Deharveng 2004) emphasized again the systematic significance of the first instar larval chaetotaxy at higher level.

We compare the primary dorsal chaetotaxy of the body among *Homidia jordanai* sp. n. and another five species of family Entomobryidae. The morphology of some primary homologous setae under Szeptycki’s nomenclature exhibits stable differentiation (mac, mic or absent) at the first instar (Table 3), though number and position of them are apparently similar. Among six species, the primary tergal chaetotaxy of *Homidia jordanai* sp. n. is closest to *Entomobryoides myrmecophila*, almost identical in number and arrangement except s-chaetae on Abd. IV. Integrating its adult features, such as elongated Abd. IV, four antennal segments and absence of scales, the genus *Homidia* apparently belongs Entomobryini sensu (Soto-Adames et al. 2008). It differs from Orchesellinae in 4 antennal segments (5–6 in the latter), elongated Abd. IV, as well as more primary setae on Abd. IV. It also can be distinguished from *Seira* and Lepidocyrid species by presence of p4 and absence of p3 (reverse in the latter) on Abd. III, and absence of E2 on Abd. IV. Among genera of Entomobryini (*Entomobrya*, *Drepanura*, *Sinella*, *Coecobrya*), adults of *Homidia* is easily separated from others in presence of dental spines, “eyebrow” on Abd. IV, and larger subapical mucronal tooth; as for first instar larvae (no dental spines), we also separate it from others by below characters: smooth labial seta E, abundant elongated s-chaetae on Abd. IV (much more than primary setae), and longer distance between area aM and pM on Abd. IV. The origin of peculiar “eyebrow” on anterior part of Abd. IV of adults couldn’t be traced by primary chaetotaxy; postembryonic development may provide key evidences to homology of enigmatic “eyebrow”.

It is still a long way to achieve the correct homology of setae in Entomobryidae. Further exploration at species level could be studied by thorough survey of more species and more characters of first instar, although first instar larvae are usually more difficult to collect than adults and subadults.

### Morphology of the “smooth” setae under SEM

Spiny setae on antennae are smooth under LM and SEM. S and ms on dorsal body smooth under LM, but not smooth under SEM (Figs 53–62, 68, 69, 72). Guard setae on labial palp, proximal setae, “smooth” setae on ventral tube, tenent hair and supraempodial seta on Tita III are also smooth under LM, but weakly ciliate under SEM (Figs 50, 51, 64–66, 74, 75). We have to carefully describe “smooth” setae in future (Chen and Christiansen 1993). Descriptions of some details (e. g. setal surface) based on LM are incomplete, and may bring confusion. SEM observation could provide fine details as a better supplementary tool for species diagnosis.

## Supplementary Material

XML Treatment for Homidia jordanai
